# The association between adverse childhood experiences and epigenetic age acceleration in the Canadian longitudinal study on aging (CLSA)

**DOI:** 10.1111/acel.13779

**Published:** 2023-01-17

**Authors:** Divya Joshi, Andrea Gonzalez, David Lin, Parminder Raina

**Affiliations:** ^1^ Department of Health Research Methods, Evidence, and Impact McMaster University Hamilton Ontario Canada; ^2^ Labarge Centre for Mobility in Aging McMaster University Hamilton Ontario Canada; ^3^ McMaster Institute for Research on Aging McMaster University Hamilton Ontario Canada; ^4^ Department of Psychiatry and Behavioral Neurosciences McMaster University Hamilton Ontario Canada; ^5^ Offord Centre for Child Studies Hamilton Ontario Canada; ^6^ Centre for Molecular Medicine and Therapeutics BC Children's Hospital Research Institute Vancouver British Columbia Canada

**Keywords:** adverse childhood experiences, CLSA, epigenetic age acceleration, GrimAge, PhenoAge

## Abstract

Research examining the association between exposure to a wide range of adverse childhood experiences (ACEs) and accelerated biological aging in older adults is limited. The purpose of this study was to examine the association of ACEs, both as a cumulative score and individual forms of adversity, with epigenetic age acceleration assessed using the DNA methylation (DNAm) GrimAge and DNAm PhenoAge epigenetic clocks in middle and older‐aged adults. This cross‐sectional study analyzed baseline and first follow‐up data on 1445 participants aged 45–85 years from the Canadian Longitudinal Study on Aging (CLSA) who provided blood samples for DNAm analysis. ACEs were assessed using a validated self‐reported questionnaire. Epigenetic age acceleration was estimated by regressing each epigenetic clock estimate on chronological age. Cumulative ACEs score was associated with higher DNAm GrimAge acceleration (β: 0.07; 95% CI: 0.02, 0.11) after adjusting for covariates. Childhood exposure to parental separation or divorce (β: 0.06; 95% CI: 0.00, 0.11) and emotional abuse (β: 0.06; 95% CI: 0.00, 0.12) were associated with higher DNAm GrimAge acceleration after adjusting for other adversities and covariates. There was no statistical association between ACEs and DNAm PhenoAge acceleration. Early life adversity may become biologically embedded and lead to premature biological aging, in relation to DNAm GrimAge, which estimates risk of mortality. Strategies that increase awareness of ACEs and promote healthy child development are needed to prevent ACEs.

## Graphical abstract

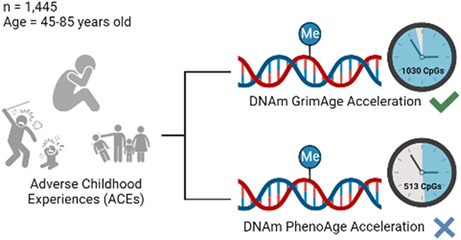

## 1 INTRODUCTION

Adverse childhood experiences (ACEs) including child maltreatment and household adversities are associated with physical, mental, and social health outcomes across the lifespan. However, the mechanisms through which ACEs become biologically embedded to impact health outcomes in later life remain poorly understood. Accumulating research suggests that epigenetic mechanisms such as DNA modification through methylation may help to explain the lasting effects of early life adversity on health (Fransquet et al., 2019; Horvath, 2013; Horvath & Levine, 2015; Levine et al., 2018; Liu et al., 2018). Studies have consistently reported associations between accelerated biological aging, where the epigenetic age is higher than the chronological age, and morbidity and mortality (Fransquet et al., 2019; Horvath, 2013; Horvath & Levine, 2015; Levine et al., 2018; Liu et al., 2018). DNA methylation‐based estimators, referred to as ‘epigenetic clocks’, are composite measures that have been developed to measure aspects of biological aging. Epigenetic clocks use specific CpG sites whose DNA methylation levels produce age estimations. Of the first‐generation clocks, the original Horvath DNA methylation (DNAm) clock included 353 CpGs across multiple cell and tissue types from children and adults and is strongly correlated with chronological age, while the Hannum DNAm clock is based on 71 CpGs and was trained solely in whole blood samples from adults (Hannum et al., 2013; Horvath, 2013). More recently, second‐generation epigenetic clocks such as the PhenoAge and GrimAge use selected CpGs associated with risk factors for disease and thus incorporated clinical biomarkers of physiological dysregulation (Levine et al., 2018; Lu et al., 2019). These newer clocks have shown improved accuracy in predicting physical functioning, time‐to‐cardiovascular disease, time‐to‐cancer, and time‐to‐mortality compared to first‐generation clocks (Levine et al., 2018; Lu et al., 2019).

Studies in children and younger adults have shown associations between exposure to ACEs including childhood exposure to sexual abuse, intimate partner violence, and poor parental mental health, and epigenetic age acceleration (Brody et al., 2016; Davis et al., 2017; Lawn et al., 2018; Nelles‐McGee et al., 2022; Simons et al., 2016; Zannas et al., 2015). Further, results from a meta‐analysis showed that childhood exposure to traumatic stress, broadly defined as the number of different types of traumatic events experienced in childhood including witnessing family violence, sexual abuse, physical abuse, or neglect occurring prior to age 18, was associated with epigenetic age acceleration; with each additional exposure to a new type of trauma associated with a 6‐month age acceleration. However, this association was only present for the Hannum DNAm Age and not for the Horvath DNAm Age (Wolf et al., 2018).

Although evidence from the literature suggests an association between exposure to childhood adversity and accelerated biological aging, most of this work has mainly examined one or two types of adversity and included socioeconomic factors as measures of ACEs as opposed to a wide range of ACEs including exposure to emotional abuse and neglect (Klopack et al., 2022). Moreover, studies have focused on the Horvath or Hannum DNAm Age algorithms with discrepant findings (Wolf et al., 2018; Zannas et al., 2015). Studies examining the impact of ACEs on the later generation epigenetic clocks in older individuals are limited. The purpose of this study was to examine the association of ACEs, both as a cumulative score as well as individual forms of adversity, with epigenetic age acceleration assessed using the DNAm GrimAge and DNAm PhenoAge in middle and older‐aged adults in Canada. We hypothesized that exposure to ACEs would be associated with epigenetic age acceleration in adults.

## 2 RESULTS

**FIGURE 1:**
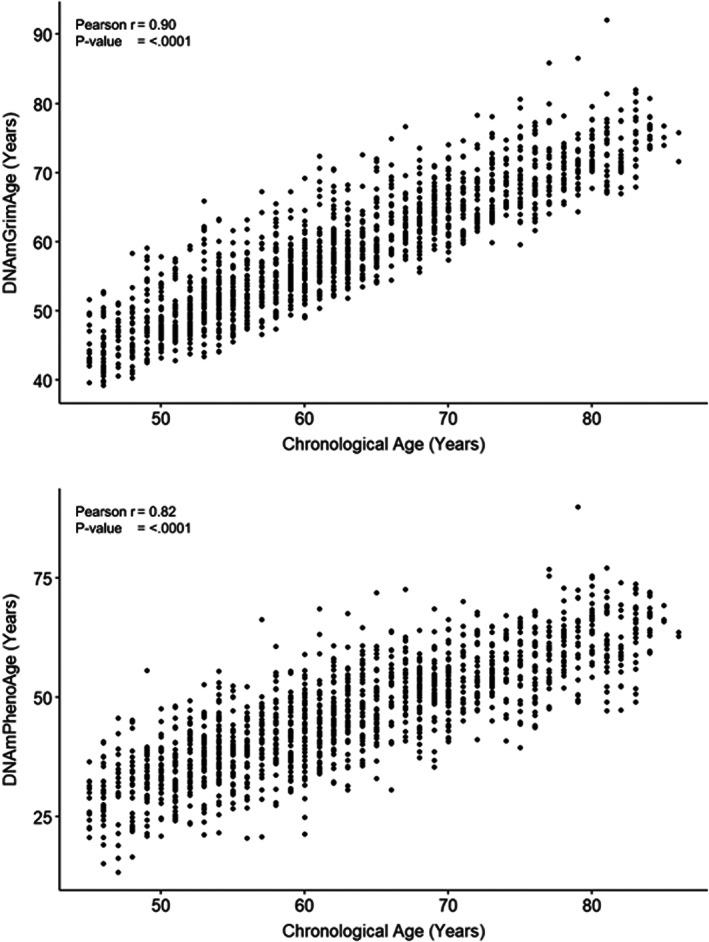
Pearson correlation between the two epigenetic age measures and chronological age

Descriptive characteristics of participants in the study sample are presented in Table 1. The average age of the participants was 63.0 years (SD: 10.3 years), 49.3% were males, majority of the participants had a post‐secondary education, and 34.9% reported a total annual income of $100,000 or more. Approximately, two‐thirds of the participants had experienced at least one ACE, 15.7% had experienced two ACEs, and 12.0% had experienced four or more ACEs. Physical abuse was the most prevalent form of ACE (28.5%), followed by living with a family member with poor mental health (23.5%), emotional abuse (23.2%), and childhood exposure to intimate partner violence (23.0%). More than half of the participants were never smokers, 29.6% participated in the recommended level of physical activity, 84.6% reported consuming high nutrition diet, and 12.7% did not drink alcohol. Overall, 40.2% of participants reported engaging in any two, and almost one‐third of participants reported engaging in at least three of four poor health behaviors. The average DNAm GrimAge was 59.1 years (SD: 9.2) and was closer to the chronological age compared to the DNAm PhenoAge (Mean: 46.2, SD: 11.7). Nevertheless, as seen in Figure 1, both epigenetic clocks correlated significantly with chronological age, with a stronger association found for DNAm GrimAge (*r* = 0.90).

**TABLE 1:** Distribution of sociodemographic factors, health behaviors, adverse childhood experiences, and epigenetic aging measures in the CLSA (*n* = 1445)

	Mean or frequency	SD or percent
Sex, *n* (%)		
Male	713	(49.3)
Female	732	(50.7)
Total annual household income, *n* (%)		
<$50,000	445	(32.6)
$50,000– <$100,000	445	(32.6)
$100,000– <$150,000	245	(17.9)
$150,000 or more	232	(17.0)
Chronological age (years), Mean (SE)	63.0	(10.3)
DNAm GrimAge (years), Mean (SE)	59.1	(9.2)
DNAm PhenoAge (years), Mean (SE)	46.2	(11.7)
ACEs, *n* (%)		
None	447	(34.2)
One	373	(28.5)
Two	205	(15.7)
Three	125	(9.6)
Four or more	157	(12.0)
Physical abuse, *n* (%)	366	(28.5)
Sexual abuse, *n* (%)	213	(15.4)
Emotional abuse, *n* (%)	299	(23.2)
Neglect, *n* (%)	53	(4.1)
Childhood exposure to intimate partner violence, *n* (%)	295	(23.0)
Death of a parent, *n* (%)	232	(17.8)
Parental divorce or separation, *n* (%)	143	(11.0)
Living with a family member with poor mental health, *n* (%)	305	(23.5)
Smoking, *n* (%)	793	(55.03)
Low physical activity, *n* (%)	964	(70.36)
High nutritional risk, *n* (%)	205	(15.39)
Alcohol consumption, *n* (%)	1229	(87.3)
Total number of poor health behaviors, *n* (%)		
None	26	(1.8)
One	284	(19.7)
Two	581	(40.2)
Three	471	(32.6)
Four	83	(5.7)

The associations between ACEs and epigenetic clocks in the unadjusted and fully adjusted models are shown in Table 2. For DNAm GrimAge, cumulative ACEs score was significantly associated with faster epigenetic age acceleration (β: 0.07; 95% CI: 0.02, 0.11) after adjusting for covariates. Further, childhood exposure to emotional abuse (β: 0.06; 95% CI: 0.00, 0.12) and parental separation or divorce was positively (β: 0.06; 95% CI: 0.00, 0.11) associated with DNAm GrimAge after adjusting for other adversities and covariates. Regression estimates for many other forms of adversity were elevated but did not reach statistical significance. Apart from emotional abuse, the associations between cumulative ACEs score and individual adversity domains, and epigenetic age acceleration assessed using the DNAm PhenoAge were not statistically significant (Table 2). Number of poor health behaviors was positively associated with acceleration of epigenetic age measured using both, DNAm GrimAge (β: 0.27; 95% CI: 0.22, 0.32) and DNAm PhenoAge (β: 0.07; 95% CI: 0.02, 0.13) clocks. Further, being a male and having lower annual income was associated with a higher epigenetic age acceleration (Table 2).

**TABLE 2:** Association between ACEs and epigenetic age acceleration measures

	DNAm GrimAge acceleration	DNAm PhenoAge acceleration
	β (95% CI)	β (95% CI)
Unadjusted models (*n* = 1307)		
Total ACEs score	0.08* (0.03, 0.13)	0.02 (−0.04, 0.07)
Adjusted model^a^ (*n* = 1240)		
Total ACEs score	0.07* (0.02, 0.11)	0.03 (−0.03, 0.08)
Number of poor health behaviors	0.27* (0.22, 0.32)	0.07* (0.02, 0.13)
Male vs. Female	0.37* (0.32, 0.41)	0.26* (0.21, 0.31)
Annual household income (REF = ≥$150,000)		
<$50,000	0.17* (0.10, 0.24)	0.05 (−0.03, 0.13)
$50,000–<$100,000	0.08* (0.01, 0.14)	0.01 (−0.06, 0.09)
$100,000–<$150,000	0.04 (−0.02, 0.32)	−0.02 (−0.08, 0.05)
Individual adversity domain models^b^ (*n* = 1182)		
Physical abuse (yes vs. no)	−0.04 (−0.10, 0.01)	−0.03 (−0.10, 0.03)
Sexual abuse (yes vs. no)	0.02 (−0.03, 0.07)	−0.01 (−0.07, 0.05)
Emotional abuse (yes vs. no)	0.06* (0.00, 0.12)	0.08* (0.01, 0.15)
Neglect (yes vs. no)	0.01 (−0.04, 0.07)	−0.04 (−0.10, 0.02)
Childhood exposure to intimate partner violence (yes vs. no)	0.04 (−0.02, 0.08)	0.03 (−0.03, 0.10)
Death of a parent (yes vs. no)	0.03 (−0.02, 0.08)	0.00 (−0.05, 0.06)
Parental separation or divorce (yes vs. no)	0.06* (0.00, 0.11)	0.04 (−0.02, 0.10)
Living with a family member with mental health issues (yes vs. no)	−0.02 (−0.07, 0.04)	−0.04 (−0.10, 0.01)

* *p*‐Value < 0.05.

^a^ Model is adjusted for sex, annual household income, and number of poor health behaviors (cigarette smoking, physical activity, alcohol consumption, and nutritional intake).

^b^ For each ACEs domain, model is adjusted for all other adversities, sex, annual household income, and number of poor health behaviors (cigarette smoking, physical activity, alcohol consumption, and nutritional intake).

The associations between cumulative ACEs score and individual components (age‐adjusted DNAm‐based surrogate markers) of the DNAm GrimAge were examined. The results showed a positive association between cumulative ACEs score and DNAm plasminogen activation inhibitor 1 (DNAm PAI‐1), DNAm beta‐2 microglobulin (DNAm B2M), and DNAm pack‐years (DNAm PACKYRS) (Table S1). Further, we explored the associations between cumulative ACEs score and individual ACEs and other epigenetic clocks including Horvath, Hannum, DunedinPoAm, and DunedinPACE. Cumulative ACEs score was positively associated with DunedinPoAm clock, but no statistically significant associations were found for the other three clocks after adjusting for covariates (Tables S2 and S3). We also explored two‐way interactions between cumulative ACEs score and number of poor health behaviors, sex, and total income and results were not statistically significant (results not reported).

## 3 DISCUSSION

The present study investigated the association of ACEs with two measures of epigenetic age acceleration in a population‐based sample of middle‐aged and older adults. The results showed that exposure to greater number of ACEs was associated with accelerated epigenetic aging, measured using the DNAm GrimAge but not DNAm PhenoAge. When examining individual forms of adversity, childhood exposure to emotional abuse and parental separation or divorce were positively associated with DNAm GrimAge acceleration after adjusting for other adversities and covariates. Further, engaging in greater number of poor health behaviors including physical inactivity, smoking, nutritional risk, and high‐risk alcohol consumption was associated with epigenetic age acceleration measured using both, DNAm GrimAge and DNAm PhenoAge clocks.

Our finding that greater number of ACEs is associated with acceleration of biological aging is largely congruent with previous studies. Early life adversity has been associated with biological age acceleration assessed using the Horvath and Hannum DNAm clocks and childhood abuse was associated with acceleration of DNAm GrimAge in younger and middle‐aged adults (Hamlat et al., 2021; Wolf et al., 2018; Zannas et al., 2015). Moreover, DNAm GrimAge has been associated with age‐related decline in various health outcomes that are also associated with ACEs. In the Irish Longitudinal Study on Ageing, DNAm GrimAge acceleration was associated with walking speed, polypharmacy, frailty, and mortality, factors that are also associated with ACEs, after adjusting for age, sex, socioeconomic, and lifestyle factors (Bellis et al., 2015; McCrory et al., 2021; Mian et al., 2022). In our study, childhood exposure to emotional abuse and parental separation or divorce were associated with a higher DNAm GrimAge acceleration. Estimates for some of the other individual forms of adversity such as childhood exposure to intimate partner violence was elevated, but did not reach statistical significance, likely due to small sample size. Nevertheless, these results provide indication of epigenetic programming from exposure to early life stress. Exposure to ACEs may induce DNAm changes that may be persistent across the life course, especially in the absence of health behavior and lifestyle interventions (Dunn et al., 2019). Guided by the stress theory, research shows that individuals who have experienced early life adversity have shown increased methylation levels, which are associated with hypothalamic–pituitary–adrenal axis dysregulation, thus suggesting the potential early life origins of adult disease (Liu & Nusslock, 2018).

Further, our results showed that co‐occurrence of poor health behaviors was associated with DNAm GrimAge and DNAm PhenoAge acceleration. Studies have shown consumption of vegetables and omega‐3 fatty acid intake were negatively associated with epigenetic age, and fat intake, insulin and glucose levels, body mass index, waist‐to‐hip ratio, and liver fat and visceral adipose tissue volume, smoking, and alcohol consumption were positively associated with epigenetic age (Ecker & Beck, 2019; Zhao et al., 2019). Research has demonstrated epigenetic aging to be associated with increased risk of frailty, time‐to‐coronary heart disease, time‐to‐cancer, and time‐to‐mortality (Li et al., 2020; Lu et al., 2019). In fact, our results showed that cumulative ACEs score was positively associated with individual components of the DNAm GrimAge including DNAm PAI‐1, DNAm B2M, and DNAm PACKYRS. PAI‐1 is an inhibitor of tissue plasminogen activator and higher PAI‐1 levels are associated with inflammation, thrombotic complications, and cardiovascular conditions (Vaughan, 2005). Likewise, B2M is a sensitive biomarker for inflammatory conditions, infections, and cancer, and is positively associated with coronary heart disease, stroke, and mortality (Bethea & Forman, 1990; Shi et al., 2021).

Strengths of this study include the availability of data on eight different forms of ACEs and the diverse epigenetic clock measures that were calculated from a relatively large, population‐based sample of middle‐aged and older adults. However, the findings of this study should be interpreted in the context of its limitations. Exposure to ACEs was assessed through retrospective self‐report and may be prone to response bias. However, the association between ACEs and health outcomes do not appear to be dependent on the timing of reporting and have been demonstrated in both prospective and retrospective studies (Karatekin & Hill, 2019). Further, exposure to ACEs may have been underestimated, given that the study sample excluded individuals residing on First Nation reserves, territories, and institutions. This was a cross‐sectional study and, as such, temporality between exposure to ACEs and epigenetic age acceleration cannot be established. Finally, the epigenetic clock measures were obtained at a single point in time. Future studies should assess epigenetic aging and other covariates using a longitudinal design so that changes in covariates can be examined in relation to the rate (deceleration or acceleration) of epigenetic aging.

## 4 CONCLUSIONS

To conclude, this study investigated the association between ACEs and diverse epigenetic age acceleration measures. For DNAm GrimAge, the results showed that exposure to greater number of different forms of ACEs and co‐occurrence of poor health behaviors were associated with accelerated biological aging. These results suggest that early life adversity may become biologically embedded and lead to premature biological aging, which may in turn increase the risk of age‐related morbidity and mortality. Therefore, strategies that increase awareness of ACEs and promote healthy child development are needed to prevent ACEs. Further, clinicians should implement trauma‐informed care to support the health needs of individuals who have experienced ACEs. Future studies should focus on identifying the intervention factors that may modify DNAm GrimAge and help reduce the impact of ACEs on cell aging and the resulting health outcomes.

## 5 METHODS

### 5.1 Study design and participants

The Canadian Longitudinal Study on Aging (CLSA) is a national, longitudinal research platform, which included participants from all 10 Canadian provinces. Using stratified random sampling, 51,338 individuals aged 45–85 years at the time of recruitment (2011–2015) were recruited from the community. These participants will be followed every 3 years until 2033 or until death or loss to follow‐up. All study participants provided data on the demographic, biological, medical, psychosocial, economic, lifestyle, and behavioral factors through either a computer‐assisted telephone survey or an in‐person home interview. Of the 51,338 participants, a subset of 30,097 participants (Comprehensive cohort) living within 25–50 km distance from one of 11 Data Collection Site (DCS) located across seven provinces were invited to visit a DCS for more detailed physical assessments and to provide blood and urine samples. From this cohort, a random sample of 1445 participants were selected for DNA methylation analysis and are included in the present study. The sample selection was made to reflect the distribution of Comprehensive cohort by region, age group, and sex. Data on epigenetic age measures and all covariates were obtained at baseline and data for ACEs were obtained at first follow‐up. The CLSA study design and methods have been described in detail by previous publications (Raina et al., 2009, 2019).

### 5.2 Study measures

#### 5.2.1 Adverse childhood experiences (ACEs)

Exposure to ACEs before the age of 16 was assessed using a 14‐item self‐reported questionnaire that was adapted from the Childhood Experiences of Violence Questionnaire (CEVQ) (Tanaka et al., 2012; Walsh et al., 2008) and the National Longitudinal Study of Adolescent to Adult Health Wave III questionnaire (Harris & Udry, 2022). Frequency and severity of childhood exposure to physical, emotional, and sexual abuse, neglect, and intimate partner violence were assessed on an ordinal scale with five response options: never, 1–2 times, 3–5 times, 6–10 times, or more than 10 times. Presence or absence of exposure to these five forms of ACEs were based on the CEVQ guidelines (Tanaka et al., 2012). Participants were identified as having experienced physical abuse if they reported being slapped on the face, head or ears, or hit or spanked with something hard 3 or more times; pushed, grabbed, or shoved, or have something thrown with the intention of hurting 3 or more times, or kicked, bit, punched, choked, burned, or physically attacked by an adult 1 or more times (Tanaka et al., 2012). Sexual abuse was identified by asking participants to report if they experienced unwanted touching or if were threatened or forced into unwanted sexual activity (Tanaka et al., 2012). Participants were identified as having experienced emotional abuse if they reported their parents or guardians swearing or saying hurtful or insulting things that made them feel like they were not wanted or loved 3 or more times. Neglect was present if participants reported that their parents, step‐parents, or guardians did not take care of their basic needs 1 or more times. Childhood exposure to intimate partner violence was present if participants witnessed verbal abuse in the home between parents or guardians 6 or more times; or if they witnessed physical abuse between parents or guardians 3 or more times (Tanaka et al., 2012). The psychometric properties of the CEVQ have been reported previously (Dube et al., 2004; Tanaka et al., 2012). Additionally, participants were asked to report on 3 other forms of ACEs including whether or not they had experienced parental divorce/separation, parental death/serious illness, or lived with a family member with mental or psychiatric illness. Participants' responses on each of the 8 individual forms of ACEs were added to calculate a total ACEs score.

#### 5.2.2 DNA methylation analysis

The methodology for profiling genome‐wide DNA methylation in peripheral blood mononuclear cells (PBMCs) in the CLSA participants has been described in the CLSA Data Support Document (David et al., 2020). Briefly, the proportion of methylation on cytoside‐guanine (CpG) nucleotide base pairs on the DNA extracted from the PBMCs was measured using the Illumina Infinium MethylationEPIC BeadChip microarrays referred to as the EPIC arrays (Illumina, CA, USA). The EPIC array quantitatively measures DNA methylation at 862,927 CpG sites and 2932 CHH sites across the genome. To obtain the DNA methylation data, we first extracted genomic DNA from the frozen PBMC samples using QIAsymphony DSP DNA Kits (Qiagen, Hilden, GE), and performed bisulfite conversion using the EZ DNA Methylation kit (Zymo, CA, USA). The resulting bisulfite‐converted DNA was processed on the EPIC arrays following manufacturer's instructions. For Quality control purposes, these raw array data were preprocessed using the GenomeStudio software (Illumina, CA, USA), which transformed the raw methylation values into beta values. The beta values range from 0 to 1 and indicate the proportion of methylation at each CpG loci present in the sample. Initial quality check of the preprocessed methylation data was carried out using the R statistical environment in RStudio (v3.6.3), where we identified and removed four samples with bisulfite‐conversion scores (as computed by the *bscon* function in the *wateRmelon* package; v1.28.0) <85%. We also removed inconsistently performing and non‐specific probes. The *wateRmelon* and *lumi* (v23.6.0) packages in R identified 29 additional outlier samples, which were also excluded from further processing procedures. Thus, the final sample included 1445 participants.

#### 5.2.3 Derivation of biological age estimates from epigenetic clocks

Epigenetic aging measures were calculated from the DNA methylation beta values using the Horvath online DNA Methylation Age calculator software (https://dnamage.genetics.ucla.edu/home). Each clock was derived using weight and beta values that were normalized using the Noob normalization approach. The DNAm GrimAge was calculated based on seven age‐related plasma biomarkers including adrenomedullin, beta‐2‐microglobulin, cystatin‐C, growth differentiation factor 15, leptin, plasminogen activator inhibitor 1, and tissue Inhibitor Metalloproteinases 1 (Lu et al., 2019). In addition, since smoking is an important risk factor for morbidity and mortality, a DNA methylation‐based estimator of smoking pack‐years was also included (Lu et al., 2019). Age and sex were also included as covariates (Lu et al., 2019). The DNAm PhenoAge was based on phenotypic age score developed from chronological age and nine clinically relevant blood biomarkers including albumin, creatinine, serum glucose, C‐reactive protein, lymphocyte percent, mean cell volume, red cell distribution width, alkaline phosphatase, and white blood cell count (Levine et al., 2018). The DNAm PhenoAge was trained to predict all‐cause mortality, and the DNAm GrimAge was trained to predict time‐to‐death. In addition, the DNAm age acceleration residuals were also estimated for each participant by regressing the biological clock estimate on chronological age.

#### 5.2.4 Covariates

Regression analyses were adjusted for sex, total annual household income (<$50,000, $50,000‐ < $100,000, $100,000‐$ < 150,000, $150,000 or more), cigarette smoking (never smoker, smoker), physical activity (adequate activity, low activity), nutritional intake (low risk, high risk), and alcohol consumption (never, occasional/regular drinker). Physical activity was assessed using the Physical Activity Scale for the Elderly (PASE) and dichotomized as meeting the World Health Organization's age specific guidelines for physical activity of at least 150 minutes of moderate‐intensity or at least 75 min of vigorous‐intensity physical activity per week (Washburn et al., 1999; World Health Organization, 2010). Nutritional intake was assessed using the “Seniors in the Community: Risk Evaluation for Eating and Nutrition (SCREEN‐II)” tool, and a previously established and validated cut‐point of <32 was used to identify participants as “high risk” (Keller et al., 2005). In the analysis, the number of poor health behaviors were summed for each participant with the score ranging from 0 to 4. These covariates were identified a priori in the literature for their association with the aging process.

#### 5.2.5 Statistical analysis

Descriptive statistics including mean and standard deviation (SD) for continuous variables and count and percentage for categorical variables were reported. Pearson correlation was used to examine the correlation between epigenetic clocks and chronological age. A multivariable ordinary least squares linear regression analysis was used to examine the association between cumulative ACEs score and epigenetic age acceleration assessed using DNAm GrimAge and DNAm PhenoAge, after adjusting for the covariates mentioned above. Multivariable linear regression models were also used to examine the association between individual forms of adversity and epigenetic age acceleration for each clock. The associations between cumulative ACEs score and individual components (age‐adjusted DNAm‐based surrogate markers) of DNAm GrimAge were also examined. We also explored associations between cumulative ACEs score and Horvath, Hannum, DunedinPoAm, and DunedinPACE. Further, we explored two‐way interactions between ACEs and number of poor health behaviors, sex, and total income. Standardized regression estimates, 95% confidence intervals, and p‐value for the unadjusted and fully adjusted models were reported. All analyses were performed on SAS version 9.4 for a two‐tail test and at a significance level of 0.05.

## AUTHORS' CONTRIBUTIONS

D.J., A.G., and P.R. were involved in the conceptualization and design of the study. D.J. and D.L. conducted the data analyses. D.J. drafted the manuscript. All authors contributed to the interpretation of the data, provided critical revisions of the manuscript, and approved the final version to be published.

## ACKNOWLEDGEMENTS

Funding for the Canadian Longitudinal Study on Aging (CLSA) is provided by the Government of Canada through the Canadian Institutes of Health Research (CIHR) under grant reference LSA 94473, the Canada Foundation for Innovation and provincial governments (Newfoundland, Nova Scotia, Quebec, Ontario, Manitoba, Alberta and British Columbia). This research has been conducted using the Baseline Comprehensive Dataset version 5.0, Follow‐up 1 Comprehensive Dataset version 3.0 under Application Number 2002018. The CLSA is led by Drs. Parminder Raina, Christina Wolfson and Susan Kirkland. Parminder Raina holds the Raymond and Margaret Labarge Chair in Optimal Aging and Knowledge Application for Optimal Aging, is the Director of the McMaster Institute for Research on Aging and the Labarge Centre for Mobility in Aging and holds a Tier 1 Canada Research Chair in Geroscience. Andrea Gonzalez holds a Tier 2 Canada Research Chair in Family Health and Preventive Interventions.

## CONFLICT OF INTEREST

The authors declare no competing interests.

## Supporting information


**Table S1.**–S3.

## Data Availability

Data are available from the Canadian Longitudinal Study on Aging (www.clsa‐elcv.ca) for researchers who meet the criteria for access to de‐identified CLSA data.
