# Supplementary material for: The association between adverse childhood experiences and epigenetic age acceleration in the Canadian longitudinal study on aging (CLSA)
Source: Aging Cell. 2023 Jan 17;22(2):e13779. doi: 10.1111/acel.13779 (PMC9924940; doi:10.1111/acel.13779)
Supplement: Supplementary file 1 — Table S1.–S3. [file ACEL-22-e13779-s001.pdf]

Supplementary Table

Table S1: Association between ACEs and components of the DNAm GrimAge[a]

	**Plasminogen activation inhibitor 1 (DNAm PAI-1)**		**Adrenomedullin levels (DNAm ADM)**		**Beta-2 microglobulin (DNAm B2M)**		**Cystatin C (DNAm Cystatin C)**	
	**β**	**95% CI**	**β**	**95% CI**	**β**	**95% CI**	**β**	**95% CI**
**Adjusted Model[a] (n=1,240)**								
Total ACEs score	0.06*	(0.01, 0.11)	0.02	(-0.02, 0.06)	0.06*	(0.00, 0.12)	-0.02	(-0.07, 0.04)
Number of poor health behaviours	0.07*	(0.02, 0.13)	0.03	(-0.01, 0.07)	-0.01	(-0.07, 0.05)	0.02	(-0.03, 0.08)
Male vs. Female	0.35*	(0.30, 0.41)	-0.69*	(-0.73, -0.64)	0.01	(-0.05, 0.06)	0.24*	(0.18, 0.29)
Annual household income (REF = ≥$150,000)								
<$50,000	-0.01	(-0.08, 0.07)	0.09*	(0.04, 0.15)	0.08*	(0.00, 0.16)	0.05	(-0.03, 0.12)
$50,000 - <$100,000	0.00	(-0.07, 0.07)	0.08*	(0.03, 0.13)	0.07	(-0.01, 0.14)	0.04	(-0.03, 0.12)
$100,000 - <$150,000	-0.02	(-0.08, 0.05)	0.04	(-0.01, 0.09)	0.01	(-0.06, 0.08)	-0.05	(-0.11, 0.02)

	**Growth differentiation factor 15 (DNAm GDF-15)**		**Leptin (DNAm Leptin)**		**Tissue inhibitor metalloproteinase 1 (DNAm TIMP-1)**		**Smoking pack-years (DNAm PACKYRS)**	
	**β**	**95% CI**	**β**	**95% CI**	**β**	**95% CI**	**β**	**95% CI**
**Adjusted Model[a] (n=1,240)**								
Total ACEs score	-0.01	(-0.06, 0.05)	0.02	(-0.01, 0.05)	0.04	(-0.01, 0.09)	0.06*	(0.01, 0.11)
Number of poor health behaviours	0.09*	(0.03, 0.15)	0.03	(-0.00, 0.06)	0.02	(-0.03, 0.08)	0.34*	(0.29, 0.39)
Male vs. Female	0.11*	(0.05, 0.17)	-0.87*	(-0.90, -0.84)	0.30*	(0.25, 0.35)	0.21*	(0.16, 0.25)
Annual household income (REF = ≥$150,000)								
<$50,000	0.06	(-0.02, 0.14)	0.01	(-0.03, 0.05)	0.06	(-0.02, 0.13)	0.18*	(0.11, 0.25)
$50,000 - <$100,000	0.01	(-0.06, 0.09)	0.04*	(0.00, 0.08)	0.01	(-0.06, 0.07)	0.06	(-0.00, 0.13)
$100,000 - <$150,000	0.02	(-0.05, 0.10)	0.01	(-0.03, 0.04)	0.01	(-0.03, 0.08)	0.06	(-0.00, 0.12)

[a]Components are age-adjusted; *p-value <0.05

Table S2: Association between ACEs and Hannum and Horvath DNAmAge acceleration measures

	Hannum DNAm Age Acceleration		Horvath DNAm Age Acceleration	
	β	95% CI	β	95% CI
**Unadjusted Models (n=1,307)**				
Total ACEs score	-0.02	(-0.08, 0.03)	0.00	(-0.06, 0.05)
**Adjusted Model[a] (n=1,240)**				
Total ACEs score	0.00	(-0.06, 0.05)	0.01	(-0.05, 0.06)
Number of poor health behaviours	0.05	(-0.01, 0.10)	0.04	(-0.02, 0.09)
Male vs. Female	0.30*	(0.25, 0.35)	0.25*	(0.19, 0.30)
Annual household income (REF = ≥$150,000)				
<$50,000	0.05	(-0.02, 0.13)	0.03	(-0.05, 0.10)
$50,000 - <$100,000	0.08*	(0.01, 0.16)	0.04	(-0.03, 0.11)
$100,000 - <$150,000	0.02	(-0.05, 0.08)	0.01	(-0.06, 0.07)
**Individual Adversity Domain Models[b] (n=1,182)**				
Physical abuse (yes vs. no)	-0.05	(-0.11, 0.01)	0.02	(-0.3, 0.08)
Sexual abuse (yes vs. no)	0.00	(-0.06, 0.06)	0.03	(-0.03, 0.09)
Emotional abuse (yes vs. no)	0.07*	(0.01, 0.14)	-0.01	(-0.08, 0.06)
Neglect (yes vs. no)	-0.03	(-0.09, 0.03)	-0.07	(-0.13, -0.01)
Childhood exposure to intimate partner violence (yes vs. no)	0.02	(-0.05, 0.09)	0.00	(-0.07, 0.06)
Death of a parent (yes vs. no)	0.01	(-0.04, 0.07)	0.08*	(0.03, 0.14)
Parental separation or divorce (yes vs. no)	-0.01	(-0.07, 0.05)	0.04	(-0.02, 0.10)
Living with a family member with mental health issues (yes vs. no)	-0.05	(-0.10, 0.01)	-0.04	(-0.10, 0.01)

[a]Model is adjusted for sex, annual household income, and number of poor health behaviours (cigarette smoking, physical activity, alcohol consumption, and nutritional intake)
[b]For each maltreatment domain, model is adjusted for all other maltreatments, sex, annual household income, and number of poor health behaviours
*p-value <0.05

Table S3: Association between ACEs and DunedinPoAm and DunedinPACE DNAm Age acceleration measures

	DNAm DunedinPoAm Acceleration		DNAm DunedinPACE Acceleration	
	**b**	**95% CI**	**b**	**95% CI**
**Unadjusted Models (n=1,307)**				
Total ACEs score	0.06*	(0.01, 0.12)	0.06*	(0.01, 0.12)
**Adjusted Model[a] (n=1,240)**				
Total ACEs score	0.05*	(0.00, 0.11)	0.05	(-0.01, 0.10)
Number of poor health behaviours	0.12*	(0.07, 0.18)	0.14*	(0.08, 0.19)
Male vs. Female	0.19*	(0.13, 0.24)	0.20*	(0.14, 0.25)
Annual household income (REF = ≥$150,000)				
<$50,000	0.13*	(0.06, 0.21)	0.15*	(0.08, 0.23)
$50,000 - <$100,000	0.04	(-0.03, 0.11)	0.01	(-0.06, 0.08)
$100,000 - <$150,000	0.12*	(0.07, 0.18)	0.14*	(0.08, 0.19)
**Individual Adversity Domain Models[b] (n=1,182)**				
Physical abuse (yes vs. no)	-0.2	(-0.09, 0.04)	-0.03	(-0.09, 0.03)
Sexual abuse (yes vs. no)	0.02	(-0.04, 0.07)	0.01	(-0.05, 0.07)
Emotional abuse (yes vs. no)	0.06	(-0.01, 0.13)	0.12*	(0.05, 0.19)
Neglect (yes vs. no)	0.02	(-0.04, 0.08)	0.02	(-0.04, 0.08)
Childhood exposure to intimate partner violence (yes vs. no)	0.01	(-0.05, 0.08)	-0.01	(-0.07, 0.06)
Death of a parent (yes vs. no)	-0.01	(-0.07, 0.04)	0.01	(-0.05, 0.06)
Parental separation or divorce (yes vs. no)	0.06*	(0.00, 0.12)	0.00	(-0.06, 0.06)
Living with a family member with mental health issues (yes vs. no)	-0.01	(-0.07, 0.05)	-0.03	(-0.09, 0.03)

[a]Model is adjusted for sex, annual household income, and number of poor health behaviours (cigarette smoking, physical activity, alcohol consumption, and nutritional intake)
[b]For each ACEs domain, model is adjusted for all other adversities, sex, annual household income, and number of poor health behaviours
*p-value <0.05
